# Analysis of China's fight against COVID-19 from the perspective of policy tools—policy capacity

**DOI:** 10.3389/fpubh.2022.951941

**Published:** 2022-09-20

**Authors:** Shuicheng Zhu, Shuaiyao Feng, Xiaoling Ning, Yiwei Zhou

**Affiliations:** ^1^Department of Public Administration, Business School, University of Shanghai for Science and Technology, Shanghai, China; ^2^College of Engineering Science and Technology, Shanghai Ocean University, Shanghai, China; ^3^Department of Information Management and Information Systems, Business School, University of Shanghai for Science and Technology, Shanghai, China

**Keywords:** China, pandemic, policy tools, policy capacity, COVID-19

## Abstract

Judging from the number of confirmed cases, deaths, cures and the time taken to restore normal social and economic order, China is undoubtedly one of the most successful countries in fighting the COVID-19 pandemic, which highlights strong policy capacity of Chinese government using policy tools to solve policy problems efficiently. Based on the policy tools theory put forward by Roy Rothwell and Walter Zegveld, this paper analyzes the specific policy tools used in the prodromal period, breakout period, chronic period and resolution period of China's COVID-19 pandemic and further summarizes three characteristics: The comprehensive use of policy tools, staging of the use of policy tools in different periods and the dominant position of supply-oriented policy tools.

## Introduction

COVID-19 is a major global disaster in this century and causes unprecedented public health emergency because of the fastest rate of transmission, the most widespread infection, and the greatest difficulty in prevention and control. It took more than 1 month to initially contain the spread of the pandemic, about 2 months to keep the daily number of newly confirmed cases in China within single digits, and about 3 months to achieve decisive results in the “defensive battles” of Wuhan and even Hubei Province. China has made great strategic achievements in fighting against the pandemic in the whole country by killing the pandemic several times in succession.

An enormous number of papers have been published to explain the reasons of China's remarkable achievements in combating the pandemic from diverse perspectives. Fu Kexin credited such achievements to China's systematic strategic plans for pandemic prevention and control, which comprises of three aspects: Firstly, committing to a people-centered approach. We should emphasis the leading roles of party organizations, party members and cadres in pandemic prevention and control. Secondly, making top-level design and institutional arrangements for pandemic prevention and control from a strategic perspective and insisting on preventing and controlling in a scientific and orderly manner in accordance with the law. Thirdly, strengthening international cooperation and building a community of human destiny in the fight against pandemics (1). Mei C, on the other hand, tookthe perspective of a policy mix consisting of traditional measures, including strict community lockdowns, cross-jurisdictional resource mobilization and sanctions by officials, which contributed to the ultimate effectiveness of China's response to the pandemic (2). From the perspective of local government governance, Professor Jianxing Yu's team brought up that Zhejiang turned out to be one of the provinces that accomplished the best outcomes in China's COVID-19 prevention and control challenges, in which social organizations assumed a critical part in the prevention and control of COVID-19. They additionally excelled at transforming the advantages of nurturing and developing social organizations into the effectiveness of pandemic prevention and control governance, thus giving full play to the different roles of social organizations in the three stages of comprehensive pandemic prevention and control. The fight against the pandemic maintains operation with both hands by paying attention to the normalization of pandemic prevention and control (3).

All the above-mentioned scholars' analyses are profound and incisive. Yet, from a public policy perspective, the great victory in China's fight against COVID-19 pandemic lies in the superb policy capabilities of Chinese government.

## What is policy capacity?

As crises and failures caused by the laissez-faire model of the market economy have grown more regular in the past two to three decades, policy capacity has become a major concern of government and academics alike (4). However, there is still no consensus among academics on the definition of policy capacity.

Scholars generally agree that the term ‘policy capacity' was first mentioned by American scholars Almond and Powell in *Comparative Politics: Systems, Process, and Policy* (1987) (5). They described policy capacity as “the ability of a political system to impact the domestic and foreign environment.” Policy capacity has become a prominent part of public policy research since the turn of the last century. Kurzer Paulette used 'policy capacity', 'administrative capacity' and 'state capacity' interchangeably in his study of the impact of regional integration on national capacity and administrative autonomy (6). When discussing globalization and democratization, Cerny has argued that 'democracies are losing the policy capacity needed to transform inputs from democratic outputs into authoritative outputs' (7). A number of experts also use 'Policy Capacity' in their work. All these experts have one thing in common: they utilize the word policy capability without explicitly defining what it means.

Those views that provide a clear definition of policy capacity fall into two broad categories: The first is described in terms of the policy process (process theory). And the second is defined in terms of resources and skills (resource skills theory).

Process theory asserts that policy capability is the ability to perform one or more, or even all, parts of the policy process. Policy capability is the ability of policy-makers to 'get it right' (8), that is, to reduce the risk of policy failure and increase the likelihood of achieving a successful solution to a problem (9). It's the ability to reduce the risk of policy failure while increasing the chances of finding effective solutions. Policy capacity is “the ability to assess the impact of policy options” (10). Policy capacity is “the ability to make informed collective choices about the allocation of scarce resources based on public purpose, particularly in setting strategic directions” (11). Policy capacity includes both the ability of governments to identify choice preferences and the ability to put those preferences into effective implementation (12). A researcher in China argued that policy capability is a key indicator of government capacity, which is a combination of six components: policy issue identification, interest integration, policy planning, policy execution, policy output, and policy evaluation (13).

Policy capacity is defined in the second category as the ability to deploy applicable resources and abilities to the policy process. “The ability to use knowledge correctly in policy formation” is defined as policy capacity (14). Personal and organizational policy capacity make up policy capacity. Personal policy capacity includes knowledge and experience, practical skills in policy-making, personality traits and so on; organizational policy capacity is concerned with things like access to evidence, personnel management, interdepartmental cooperation, and leadership, etc. (15). Some scholars argued that integrity, rule of law, meritocracy, social trust and legitimacy as key elements of policy capacity (16, 17). Some scholars viewed that policy competence is a mixture of personal policy analysis and political skills (18). A number of experts suggested that policy capability consists of nine interrelated capabilities. These nine capabilities are individual analytical capability, individual operational capability, individual political capability, organizational analytical capability, organizational operational capability, organizational political capability, systemic analytical capability, systemic operational capability, and systemic political capability (19, 20). A scholar argued that policy capacity is the ability of the state to conduct theoretical research, policy analysis and communication in the policy process (21).

There are three main approaches to measuring policy capacity: The outcome approach emphasizes the measurement of the actual effects of policy outcomes. The output approach advocates measuring the quantity and quality of policy supply. And the input approach focuses on the dynamics of the input factors in the policy development process (22). Although some scholars disagree with the outcome path, arguing that policy outcomes are frequently evaluated with social and political biases and that the evaluation criteria may change over time (23). In the context of the fight against COVID-19, it is not surprising that the outcome pathway has been used as a tool to evaluate policy outcomes. The outcome pathway, on the other hand, may be utilized to analyze the important public health event of the battle against COVID-19 outbreak. Policy outcomes in the struggle against the pandemic may be quantified in a variety of objectively measurable aspects, including the number of confirmed cases, fatalities, cures, and the time it takes to return to socioeconomic normality. There is no doubt that the effectiveness of China's policies as measured by these dimensions has been remarkable. Desmond Tan, the Director-General of WHO, has praised China's response to the pandemic on numerous occasions. According to the Clove Global COVID-19 Map 2021, which was released on August 18, the total number of people diagnosed with COVID-19 in the United States has surpassed 37 million, accounting for more than 10% of the total population, with more than 620,000 deaths and about 30 million cures. China, the first country to identify and report the outbreak of the COVID-19, has diagnosed a cumulative total of about 120,000 people, representing only 0.008% of China's total population. The cumulative death toll is only 0.5 million and a cumulative cure toll of over 110,000, which maximizes the protection of Chinese citizens' lives and restores economic and social normality only about 3 months after the outbreak.

## Policy tools: A powerful tool for demonstrating policy capacity

Essentially, outcome path policy capability is the extent to which policy agents are able to solve a policy problem or achieve a policy objective, which in turn depends on the combined use of different types of policy tools. Policy tools are, in short, 'the specific means and methods used to solve a social problem or achieve a policy objective' (24). The choice of policy tools has a significant impact on the achievement of stated policy objectives and the resolution of potential or apparent policy problems in the state and social governance process.

**Figure 1:**
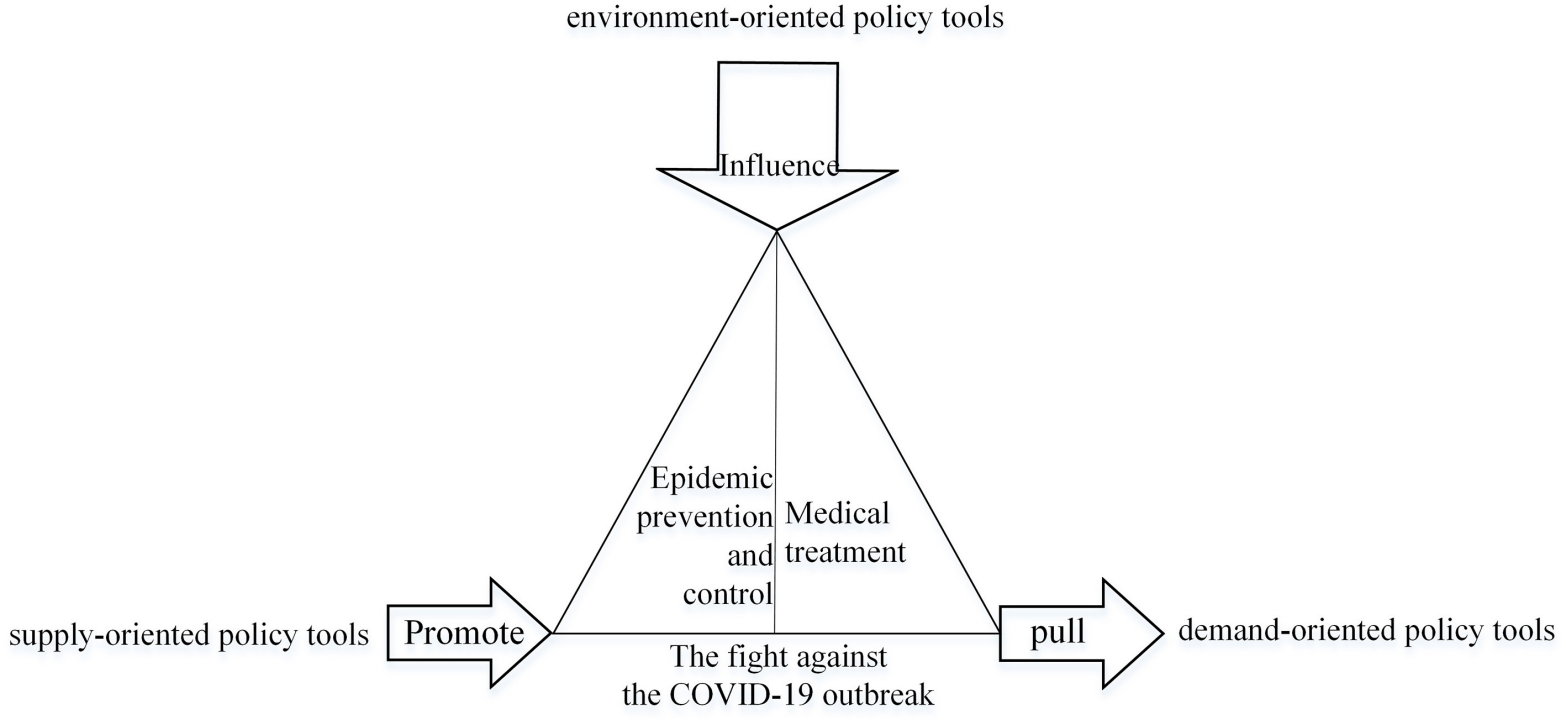
The role of different types of policy tools in the fight against the COVID-19 outbreak in China.

Two American scholars classify policy tools into three categories: supply-oriented, environment-oriented and demand-oriented (25). These three forms of policy tools are more suitable for analyzing the types of policy tools used in China's fight against the pandemic because they are fully concerned with the logic of action between policy implementation subjects and target groups. Supply-oriented policy tools refer to public policy subjects relying on their power to take the initiative to provide various human, financial, information, technology and other relevant elements. Demand-oriented policy tools are those that rely on market mechanisms to entice enterprises, social organizations, the general public, and other actors to participate in the process of policy implementation through government-led initiatives. Environment-oriented policy tools are laws, regulations, and public policies that are implemented to establish a favorable social environment and provide fertile ground for policy implementation. “There are two fronts in the fight against diseases,” General Secretary Xi Jinping said during his visit to Wuhan, Hubei Province, “one is the hospital position for saving lives and aiding the injured, and the other is the community position for prevention and control.” The entire pandemic response can be divided into two main areas: medical treatment and pandemic prevention and control. Supply and demand-oriented policy tools have a direct role in driving and pulling China's response to the pandemic, while environment-oriented policy tools have a more indirect role (Figure 1).

The policy tools in China's anti-pandemic process can be classified and described as follows, based on Roy Rothwell and Walter Zegveld's methodology of classifying policy tools and taking the 'two fronts' argument (Table 1) into account.

**Table 1:** The classification and description of policy tools in the fight against the COVID-19 in China.

**Types of policy tools**	**Types of policy sub-tools**	**Description of the tool**
**Supply-oriented policy tools**	Fiscal and financial support	This refers to the government's assistance in the form of different subsidies, tax cuts, and cash allocations. These measures include improving medical infrastructure, boosting investment in medicine and vaccine research and development, treating patients, immunizations, nucleic acid testing, company and individual tax and fee savings.
	Information support	This refers to the central and local governments, through a combination of on-site and online releases, timely information on the pandemic situation, scientific research, and clarification of rumors of domestic and international concern. Information related to the pandemic situation mainly includes new confirmed cases, new cured and discharged cases, existing confirmed cases, existing suspected cases, new deaths, and the movement trajectory of infected persons and close contacts.
	Social control	This refers to tight traffic control, personnel and premises control, and other measures used to disrupt the pandemic transmission chain.
	Personnel coordination	This refers to the reorganization of human resources required for hospital treatment and community prevention and control to meet the pandemic prevention and control's needs, such as the formation of a new command and control command organization on the basis of the existing organizational structure, the coordination of national or regional medical and nursing staff, and the implementation of cross-regional and cross-unit assistance by volunteer teams.
	Material deployment	This refers to the government's general coordination of local subsistence, pandemic prevention, and security supplies to aid in the pandemic's struggle.
**Demand-oriented policy tools**	Expert involvement	This implies that the government takes full advantage of medical professionals' recommendations when making policy decisions to guarantee that policies are scientifically sound.
	Public awareness mobilization	This refers to encouraging and guiding the entire population to participate in the fight against the pandemic through political mobilization, publicizing the deeds of famous people, and other means, as well as increasing the motivation and initiative of various groups to stop the virus from spreading.
	Public-private partnerships	This refers to the government and corporations or societal forces collaborating to provide public goods or services relevant to pandemic prevention and control.
**Environment-oriented policy tools**	Laws and regulations	This refers to various texts, other than technical regulations, issued by the body of laws and relevant policies related to pandemic prevention and control.
	Technical regulations	This refers to the guiding documents issued by the relevant authorities for the treatment and prevention and control of the new coronavirus pneumonia, including the basics of the prevention and control of the pandemic, the “Novel Coronavirus Pneumonia Treatment Plan” and the “Novel Coronavirus Pneumonia Prevention and Control Plan.”
	Target planning	This refers to the coordinated planning of objectives and tasks in order to promote pandemic prevention and control, as well as the adjustment of those objectives and tasks as needed in response to the progress of pandemic prevention and control in order to improve the effectiveness of the pandemic fight.

Source: Author's own production.

## The combined use of different types of policy tools in the fight against the pandemic in China

### Analysis of the use of policy tools based on the life cycle of a public crisis

Since specific policy objectives in the pandemic response process are constantly modified to the changing scenario, policy tool selection and implementation shall likewise be optimized in light of the changing situation. As a result, it is vital to examine the specific use of policy tool combinations in China's pandemic response in the framework of crisis life cycle theory. This paper embraces the four-stage theory of the life cycle of a public crisis: prodromal period, breakout period, chronic period and resolution period (26). The crisis prodromal period refers to the stage when a crisis has not yet manifested, yet the potential triggers for a crisis have already emerged and the crisis is in its gestation phase. On the off chance that individuals can precisely recognize the variables that trigger an emergency and go to fitting control lengths, they can nip the crisis in the bud. The crisis breakout period is the phase when the multiple components that trigger a crisis have reached a critical level and are activated by a single “factor.” The scope and intensity of the crisis keeps on rising. The crisis chronic period means the stage when the related organization has done whatever it takes to research the emergency, go to lengths to control the scope and extent of the crisis and carry out recovery work. But with the developing of crisis, the unfavorable impacts of the crisis goes on spreading. In the resolution period, the crisis has been effectively contained, the apparent hardships produced by the crisis have been generally rectified, and the burden on crisis responders has been significantly eased, but there is still a need to prevent the crisis from reoccurring. The crisis response process in China is separated into four phases in the following section based on Fink's crisis life cycle theory. And the deployment of policy tools in each step is depicted exhaustively.

#### The crisis prodromal period (2019.12.12~2020.1.19)

The first case of unexplained pneumonia was reported in Wuhan, Hubei Province, on 12 December 2019 (27). The Wuhan Municipal Health Commission reported on 12 January 2020, that a total of 41 cases of pneumonia caused by a new coronavirus infection had been discovered, with no linked cases perceived among close contacts. The presence of human-to-human transmission of the new coronavirus was clarified by a high-level expert committee from the National Health Commission of the People's Republic of China late on January 19. If the nature of human-to-human transmission was accurately assessed and effective treatment and prevention measures were taken during the crisis prodromal period, the spread of the outbreak should have been contained in the very beginning. However, as the new coronavirus pneumonia is a new pandemic with unknown etiology, existing medical technology is unable to make a swift and accurate judgment in the near term, therefore the new coronavirus's human-to-human nature could not be determined until January 20.

A combination of environmental, supply-oriented and demand-oriented policy tools were used during this period. During the crisis prodromal phase, the policy objective was to identify new cases of pneumonia in Wuhan as having human-to-human transmission characteristics and to implement the required treatment, prevention, and control measures at the same time. The environmental policy tools used to attain this policy goal are primarily laws and regulations, technical regulations, and information assistance. The legal framework is based on existing laws and regulations for dealing with serious public health events like the COVID-19 pandemic. China's Infectious Disease Prevention and Control Law (IDPC), the Emergency Regulations for Public Health Emergencies, the National Emergency Response Plan for Public Health Emergencies, and the Emergency Response Act were all previously in effect. Information support is mainly reflected in the government's initiative to announce the development of the pandemic to the society and to regulate and steer public opinion to maintain social stability. Since December 31, 2019, the government of Wuhan has begun providing information on the pandemic, and will progressively increase the frequency of information release.

The main technical specifications promulgated for the prevention and control of the pandemic are: (1) The preventive measures and warnings to seek medical consultation when symptoms appear, as mentioned by the Wuhan Municipal Health Commission in several pneumonia outbreak bulletins. (2) National Health Commission of the People's Republic of China (NHC) formulated and issued the “Three Early Programs” for the prevention and control of viral pneumonia of unknown origin, as well as the first and second editions of the Diagnosis and Treatment of Pneumonia in Novel Coronavirus Infection. (3) NHC and Wuhan Municipal Health Commission have developed a number of documents, including the Diagnosis and Treatment Protocol for Viral Pneumonia of Unknown Cause (Trial), Medical Treatment Manual for Viral Pneumonia of Unknown Cause (Trial), Diagnosis and Treatment Protocol for Pneumonia of Novel Coronavirus Infection (Trial).

During the crisis prodromal stage, professionals are mostly involved in scientific patient therapy and determining whether there is human-to-human transmission. In this regard, NHC and Wuhan Municipal Health Commission have repeatedly organized experts to study and evaluate the disease, treatment regression, epidemiological investigation and laboratory testing. On January 7, the first novel coronavirus strain was successfully isolated by the Chinese Center for Disease Control and Prevention (CCDC). The preliminary test kit was created on January 10 by China CDC and Wuhan Institute of Virology of the Chinese Academy of Sciences. On the same day, the genome of the novel coronavirus was successfully deciphered by the Institute of Biomedical Sciences Fudan University, paving the door for future pandemic prevention and control. After a thorough investigation, the high-level expert committee convened by NHC unambiguously suggested the presence of “human-to-human” transmission of the novel coronavirus on the evening of January 19^th^.

Personnel coordination was primarily manifested in the formation of new organizations and the use of existing bureaucratic organizational methods to boost the pandemic's prevention and control. The newly established organizations include: (1) NHC and the Wuhan Municipal Health Commission, respectively established an expert group to study the pandemic. (2) NHC dispatched separate pandemic prevention and control working groups and supervisory teams to the localities. (3) NHC set up the leading group for outbreak response and handling. Initiatives to strengthen the prevention and control of the pandemic using the existing bureaucratic organizational system include: (1) Several government working meetings on pandemic prevention and control were held from top to bottom to put forward requirements for the prevention and control of the pandemic. (2) The existing national, provincial, prefectural, municipal and county-level direct reporting systems for public health emergencies were used to report cases around the country.

The social control policies used during this period were: (1) Issue the “Announcement on Market Closure and Remediation” by the Jianghan District Market Supervision Administration and the Jianghan District Health and Health Bureau on January 1, 2020 to carry out environmental and sanitary remediation of the seafood market in Wuhan. (2) Rectify live animal markets and pedlars' markets and various types of premises. (3) Set up temperature testing points and investigation points at Wuhan airport, railway station, long-distance bus station and passenger terminal since from January 14, 2020 to strengthen temperature testing of people leaving Wuhan. (4) Intensify “daily disinfection” and “ventilation per shift” for public transport in the city. (5) Reduce the number of large public gatherings in accordance with the principle of “not holding events unless they are necessary.”

#### The crisis breakout period (2020.1.20~2020.2.18)

The crisis outbreak period began with the confirmation of “human-to-human” transmission of COVID-19 and ended on February 18. It's the first time the number of new hospital discharges exceeded the number of new confirmed cases, both nationwide and in Wuhan (28). The policy goal for this phase was to conduct a countrywide outbreak control effort to stop the disease from spreading.

Supply-oriented policy tools at this stage include fiscal and financial support, social control, personnel coordination, material deployment and information support. Fiscal and financial support was used to ensure the supply of key medical supplies and daily necessities for the prevention and control of the pandemic, and to support the prevention and control of the pandemic and the development of enterprises in related industries. Up to 13 February 2020, a total of RMB 80.55 billion has been arranged at all levels of finance for prevention and control, with actual expenditure of RMB 41 billion (29). The social control policy is reflected in: (1) Starting at 10:00 AM on January 23, Wuhan was put into lockdown. All residential areas in Wuhan were under closed management. (2) Beginning on February 2, Wuhan was the first city to start the “four categories of people,” in accordance with the “four shoulds” requirement. “^1^(3) On February 17, Wuhan launched a three-day centralized netting survey to promote the implementation of the “Five Hundred Percent”^2^. (4) In provinces and cities with large population inflows, the movement of people is effectively controlled in accordance with the requirements of “joint prevention and control, group prevention and control.”

Hubei Province, particularly Wuhan, was earnestly in lacking of medical and living commodities, as well as infected medical personnel and a medical system on the point of collapse due to the pandemic's abrupt emergence. Under the central government's unified arrangements, nationwide co-ordination of personnel and deployment of supplies were also carried out in a steady profression, including: (1) Mobilizing the military to support Hubei especially Wuhan. (2) Establishing provincial counterpart support mechanism, with 19 provinces supporting 16 cities, states and counties in Hubei Province, excluding Wuhan, in the form of one province being responsible for one city. (3) The joint Prevention and Control Mechanism of the State Council coordinating the dispatch of medical N95 masks in Hubei Province. (4) Concentrating superior medical resources and technical strength to treat patients, especially the severe cases, to increase the treatment rate. (5) Mobilizing a wide range of civil servants, enterprise and public institution around the country to sink to the front line of community prevention and control. Simultaneously, the organizational system for the prevention and control of the pandemic was further improved: (1) The Central Government of China set up a leading group to deal with the pandemic and at the same time set up a pandemic prevention and control steering group to guide the work in Hubei and other areas with serious pandemics. (2) NHC took the lead in setting up a joint prevention and control mechanism to deal with the pneumonia pandemic caused by the new coronavirus, with working groups for pandemic prevention and control, medical treatment, scientific research and research, publicity, foreign affairs, logistics and forward work. (3) At the local level, provincial (city) level, city/county level and community-level emergency command organizations were set up.

Information support during the crisis outbreak period has expanded in terms of both content and distribution channels compared to the previous phase. Through multiple channels such as press conferences, official statements, and mainstream media, the central and local governments provided frequent information on the pandemic's development, the whereabouts of confirmed cases, and scientific knowledge on outbreak prevention and management. Demand-oriented policy tools included public awareness mobilization and public-private partnerships, in addition to the extensive involvement of experts during this phase. The experts' expertise and wisdom are essential for the treatment of diagnosed patients and the specific measures to prevent the spread of the disease internally and to prevent its importation externally, and policy recommendations such as the “closure of Wuhan” and “one province in charge of one city” were advanced by experts and scholars and adopted by the central government. During this time, the mobilization was primarily focused on three areas: initially, the mobilization of members of the party and government system, treating pandemic prevention and control as a major political task. Secondly, the main leaders of the Hubei Provincial Government and Wuhan Municipal Government were adjusted, and CPC members and cadres who did not take charge, did not act, or neglected their duties were seriously held accountable, while those who dared to take charge and were conscientious and responsible were strongly commended and boldly used. The third is the mobilization of the general public. Through press conferences, television, newspapers, new media, radio, drones, slogans, and other slogans, the public was urged to join the fight against the pandemic and conduct a “people's war” against it. The central and local governments provide subsidized interest rate support for loans to key enterprises involved in pandemic prevention and control, such as health and pandemic prevention, vaccine production and testing, pharmaceutical products and medical equipment, in order to speed up technological transformation, expand production capacity, prevent price inflation, and continuously improve efficiencies. In addition, through the cooperation between the government and enterprises, the “Yuhang Health Code” has been developed, and the “Yuhang Health Code” has been transferred to the “Hangzhou Health Code” and then to the “National Health Code.” This has made cross-regional travel across the country easier.

The following are the key characteristics of environment-oriented policy instruments in comparison to the preceding period: (1) The prevention and control of the pandemic in Hubei Province is a top priority at the moment, and the principle of “concentrating on patients, experts, resources and treatment” is being implemented in an effort to increase the admission and cure rates while reducing the infection and ward rates. (2) On January 20, the State Council designated the new coronavirus pneumonia as a Class B infectious disease under the IDPC and adopted measures for the management of Class A infectious diseases. (3) NHC and six other departments issued the Notice on Strictly Preventing the Transmission of Novel Coronavirus Infected Pneumonia through Transportation. (4) From the 23^rd^ to the 29^th^ of January, provinces launched the provincial-level response to serious public health emergencies. (5) Extension of the Chinese New Year holiday and postponement of the opening of universities, colleges, primary and secondary schools and kindergartens in various regions. (6) The National Medical Product Administration has given emergency permission to four new coronavirus diagnostic kits developed by four companies.

The technical specifications applied during the outbreak period include: (1) The Technical Guidelines on the Selection and Use of Masks for Prevention of Novel Coronavirus Infection for Different Populations issued by the Joint Prevention and Control Mechanism of the State Council, and six public prevention guidelines issued by the National Health and Welfare Commission for general use, travel, family, public places, public transport and home observation. (2) NHC's Pneumonia with the Guidelines on the Novel Coronavirus-Infected Pneumonia Diagnosis and Treatment, the fifth edition of the revised Guidelines on the Novel Coronavirus Pneumonia Diagnosis and Treatment, the second, third and fourth editions of the Pneumonia with Novel Coronavirus Prevention and Control guidelines^3^, the Notice on the Issuance of Guidelines for Emergency Psychological Crisis Intervention in Pneumonia with Novel Coronavirus-Infection, and the Centralized Treatment Plan for Severe Pneumonia Patients Infected by Novel Coronavirus by NHC, etc.

#### The crisis chronic period (2020.2.19~2020.4.28)

The crisis chronic period is the period when the impact of the crisis outbreak continues. At this time, crisis management has achieved certain results. If handled properly, the ongoing time would be significantly abbreviated and the adverse consequence brought about by the crisis will be extraordinarily diminished. The key policy goals of this phase are to put in place precise and graded preventative and control measures, as well as to restore the order of social development.

Social control policies during the crisis chronic period have encountered huge changes. Local governments reduced the intensity of reaction to major public health emergencies at the provincial level starting on February 21, and progressively removed access restrictions.^4^ Simultaneously, nationwide community grids were used to conduct carpet checks on the “four categories of people” (confirmed patients, suspected patients, fever patients who cannot rule out the possibility of infection, and close contacts of confirmed patients), track close contact itineraries, classify and control premises, and strictly enforce the quarantine system. Except for Hubei Province and Beijing, all major highway chokepoints had been opened by February 24. And entrance and departure quarantines had been reinforced on February 25 to prevent the illness from spreading across borders. Wuhan relaxed the restrictions on corridors leading away from the city on April 8. With the resumption of work and school, the usage of health codes has grown in popularity, as “external prevention of importation and internal prevention of spread” has emerged as a key method for containing the pandemic.

Financial allocations were maintained to be used for pandemic prevention and control, as well as the execution of tax and loan interest reductions for qualifying firms and people, during the crisis chronic stage. The national financial provisions for the prevention and control of the pandemic had reached RMB 116.9 billion as of March 13 (30). Important technical regulations involving the prevention and control of the pandemic include: (1) Leading Group of the CPC Central Committee for novel coronavirus Prevention and control issued the Notice on Further Improving the Prevention and Control of the COVID-19 Pandemic in Key Places and Units and for Key People, the Norms for the Management of Asymptomatic Infected Persons with the COVID-19, the Guidelines on Measures for the Prevention and Control of the Pandemic in Enterprises and Institutions Resuming Work and Production in Different Risk Areas of the Country, and the Guidance on the Regular COVID-19 Epidemic Prevention and Control. (2) The Circular on Prevention and Control of the COVID-19 Pandemic in a Scientific and Precise Manner in Accordance with the Law^5^, the Guidelines on Measures for Prevention and Control of the Pandemic in Enterprises and Institutions Resuming Work and Production, the Circular on Further Implementation of Zoning and Grading Differentiated Prevention and Control Strategies, and the Guidelines on Measures for Prevention and Control of the Pandemic in Enterprises and Institutions Resuming Work and Production in Different Risk Areas of the Country, issued by the Joint Prevention and Control Mechanism of the State Council. (3) NHC issued the fifth and sixth COVID-19 Disease Prevention and Control Guideline and the sixth and seventh COVID-19 disease Diagnosis and Treatment Guideline.

Environment-oriented policy tools relevant to target planning are as follows: (1) The central government has put forward targeted policy objectives in response to changes in the pandemic prevention and control situation, including “Continuing to focus its efforts and resources on strengthening the prevention and control of the pandemic in Hubei Province and Wuhan City,” “Improving differentiated prevention and control strategies, strengthening prevention and control in vulnerable areas, and doing its utmost to prevent and control the pandemic in Beijing. Establishing an economic and social operation order compatible with the prevention and control of the pandemic,” “Strengthening scientific research on pneumonia prevention and control,” “Accelerating the research and development of vaccines with multiple technical routes,” “Focus the prevention and control of the pandemic focus on external prevention of importation and internal prevention of rebound,” “Support the orderly resumption of work and production in Hubei,” “On the premise of strictly doing a good job in the prevention and control of the pandemic, promoting the speed and expansion of resumption of work and production in a strong and orderly manner,” etc. (2) Leading Group of the CPC Central Committee for novel coronavirus Prevention and control promulgated the Implementation Opinions on Strengthening Measures to Stabilize Employment in Response to the Impact of the COVID-19 outbreak and the Guidance on Actively and Orderly Promoting the Resumption of Work and Production while Effectively Preventing and Controlling the COVID-19 outbreak. (3) The State Council executive meeting successively studied and deployed “Promote transportation, express delivery and other logistics to speed up the resumption of work and production to provide strong support for the prevention and control of the pandemic, smooth economic circulation and meet people's livelihood needs,” “Better play the role of special refinancing and rediscounting policies to support the prevention and control of the pandemic and the development of enterprises to alleviate difficulties,” “Promote the manufacturing and distribution industries to actively and orderly resume work and production measures while doing a good job in pandemic prevention and control,” etc. (4) The General Office of the State Council issued the Implementation Opinions on Strengthening Employment Stabilization Initiatives in Response to the Impact of the COVID-19 outbreak.

Mobilization at this stage was emphasized by General Secretary Xi Jinping's mobilization on February 23 *via* a video message broadcast directly to 170,000 CPC cadres around the country, urging for an unwavering emphasis on the pandemic's prevention and control.

#### The crisis resolution period (2021.4.29~)

The major crisis situation of the preceding phase has been nearly defeated during the resolution period, despite the fact that there is still the chance of resurgence. Preventing the crisis from resurfacing is a critical problem that must be handled during this time. China's COVID-19 outbreak control has been in the normalization phase, or receding crisis stage, since April 29.

The target planning for the crisis resolution period is mainly reflected in the different policy objectives put forward by the central government at different points in time, such as “grasping the normalized pandemic prevention and control, improving the measures of 'external prevention of importation and internal prevention of rebound' according to the situation at the time… and fully promote the resumption of work and production and the restoration of normal economic and social order in the context of normalized pandemic prevention and control,” “grasp the prevention and control of pandemics in key areas and groups, and strengthen the prevention and control of imported risks in a targeted manner,” “strengthen key areas and places to prevent internal rebound work, … strengthen external prevention of imported key areas and weak links,” etc. The Joint Prevention and Control Mechanism of the State Council has issued the Guidance on the Regular Prevention and Control of the COVID-19 Outbreak, the Implementation Advice on Accelerating the Nucleic Acid Testing of the COVID-19, the Circular on the Role of Sentinel Sites in Medical Institutions for the Regular Prevention and Control of the Pandemic, the Circular on Comprehensive and Precise Environmental Hygiene and Disinfection Work, the Notice on Promoting Orderly Movement of Personnel through Precise Health Management, and the Notice on Further Accelerating the Capacity of Medical Institutions for Nucleic Acid Testing of COVID-19.

In terms of social control, as the pandemic situation has taken a turn for the better, all provinces reduced their emergency response levels to Level 2 or below on May 1, with the exception of Hubei Province, which went from Level 1 to Level 2 on May 2 with a health code but no quarantine. The danger level was dynamically modified in real time in response to the outbreak that arose in numerous regions during the crisis resolution period, and control measures were performed at several levels.

The main technical regulations involved since the beginning of the crisis resolution period are: (1) The Protection Guidelines Related to the Regular Prevention and Control of the COVID-19 Pandemic in Key Units and Places in Key Areas during the Summer in Low-risk Areas, the Work Plan to Further Promote the Capacity Building of New Coronavirus Nucleic Acid Testing and the seventh and eighth COVID-19 Disease Prevention and Control Guideline issued by the Joint Prevention and Control Mechanism of the State Council. (2) The Eighth (including the revised version) COVID-19 Disease Diagnosis and Treatment Guideline issued by NHC.

### Analysis of the characteristics of the employment of policy tools in the fight against the pandemic in China

The preceding analysis reveals three main features of the use of policy tools in China's fight against the pandemic.

#### The comprehensive use of policy tools

Both the dimension of the kind of instrument and the degree of the sub-instrument demonstrate the integrated usage of policy tools. Both types of policy tools and individual policy sub-tools have their own strengths and weaknesses. It is only when they are effectively integrated that they may form a “co-temporal interaction” and better address real-world policy challenges, allowing policy skills to be exhibited. As mentioned earlier, China has used a combination of supply-oriented, demand-oriented and environment-oriented policy tools in all three stages except for the crisis prodromal period, as well as a mix of different sub-policy tools from the three types of policy tools. For example, policy tools include fiscal and financial support, social control and personnel coordination in the supply-oriented policy tools, expert participation and social mobilization in the demand-oriented policy tools, and target planning, technical planning, laws and policies in the environment-oriented policy tools.

#### The phasic characteristics of the use of policy tools

Although multiple types of policy tools and their specific policy sub-tools were utilized in conjunction at different periods during the COVID-19 outbreak's evolution, their utilization remained inconsistent. Early on in the pandemic, containment was mostly reliant on existing infectious disease laws and procedures, with the disease being treated as a typical pneumonia infection and no strong quarantine measures in place. During the pandemic phase, the presence of specialists was critical. All three types of policy measures, including supply-oriented, demand-oriented and environmental-oriented policy tools-, have been deployed after the pandemic reached the outbreak, chronic, and resolution stages, albeit with different emphases at various periods. Furthermore, within each kind, the employment of policy sub-tools differed. In the instance of supply-oriented social control policy sub-tools, there was an exterior “city lockdown” and an internal “district lockdown” in Wuhan during the peak of the pandemic, as well as stringent limits on cross-border movement in other provinces and cities. As the pandemic was well managed across the country, social control strategies were adjusted, going from a county (city, district) level of danger to a street, township, or even community level of risk.

#### The dominance of supply-oriented policy tools

The synergy between the two fronts of “hospital-based life-saving” and “community-based prevention and control” was the key to China's success in fighting the COVID-19 pandemic. To address the difficulty of treatingCOVID-19 patients at the start of the pandemic in Wuhan, China immediately erected “Huoshenshan Hospital,” “Leishenshan Hospital,” and the mobile cabin hospital, all of which are centralized treatment facilities for COVID-19 patients. Medical and nursing personnel were arranged from the military and autonomous regions of the country to assist Wuhan, and various pandemic prevention materials and living supplies were delivered to the Hubei area. Arrangements were made to have the country's superior scientific research forces speed up the research, development, and use of medications, vaccines, and testing reagents. After the pandemic has entered the resolution phase, the mobilization of personnel and materials remains indispensable. Volunteers, community workers, public security police, customs officers, grassroots cadres, and sinking cadres stood vigil inspecting individuals, preventing and controlling diseases, and advocating policies to defend the “front gate” of the pandemic in 650,000 urban and rural communities around the country. More than 400 billion Yuan will be invested in pandemic prevention and control at all levels of finance by the end of 2020 (31). Strengthening community prevention and control, as well as immunization, is the strongest treatment for combating the pandemic at numerous stages over the resolution phase. On the mainland, a total of 200,391,400 doses of the new crown vaccination had been distributed as of August 26, 2021 (32). Supply-oriented policy tools include the above-mentioned government financial investment, personnel coordination, material dispatch, community control, and free immunization.

## Discussion

From its initial localized outbreak, the COVID-19 pandemic has now spread globally, with huge impacts on life safety, economy, society and public health. These effects frequently interact with one another and can contribute to more extensive and harmful effects if they are combined. First and foremost is life safety. Albeit the COVID-19 pandemic isn't so lethal as many other infectious diseases, it is proving to be far more dangerous because in places with severe outbreaks, the infection fatality ratio is between 1 and 3% (33). And as we frequently say, “life is priceless.” The diversifying effects on the world economy is the second. The global macro-economy has been severely impacted since the epidemic, among other repercussions being a fall in financial markets, slower manufacturing of goods, disruptions to supply chains, and economic contraction and revenue loss (34). The economic impact of the COVID-19 outbreak is fundamentally different from the economic impact of the financial crisis, which is more of a financial flaw, whereas there is no certainty as to when the COVID-19 pandemic crisis will end and the economy will rebound (35). The rise in inequality is the third and, to all of us and, most concerning point. Although the COVID-19 pandemic outbreak has affected people all throughout the world, regardless of their income or poverty, the impact on the poor has undoubtedly been fatal. Indigenous, Latino, Pacific Islander and Black Americans all had much higher COVID-19 mortality rates than Whites or Asian Americans, which demonstrates the strong racial character of the New Coronavirus mortality rate in the United States (36). In addition to this, the United Nations' Financing for Sustainable Development Report 2021 states that the COVID-19 epidemic could result in the loss of 114 million units worldwide, with approximately 121 million people falling into extreme poverty (37). According to a Chinese study, particularly impoverished households are more likely to believe they will experience poverty during the pandemic (38).

Much of the world is still plagued by the COVID-19 epidemic as of the time this article was finished. The New Crown pneumonia pandemic has made the shocking discovery that even developed countries lack adequate preparedness for emerging diseases. We can identify and then consider the question, why is the COVID-19 epidemic so “uncontrolled” and “deadly” in some countries and not in others? Initially, although it shocks us greatly, we must acknowledge that public health systems in many countries are overburdened. More than 2 years after the outbreak of the COVID-19 epidemic, many countries are still unable to withstand the sudden outbreak, including Japan, which recently reported the highest number of confirmed cases of COVID-19 globally for four consecutive weeks. In a joint statement, fourteen Japanese medical institutions, including the Kyoto University Medical School Hospital, stated that the spread of the seventh wave of COVID-19 has reached “catastrophic levels,” the health care system is collapsing. The public health systems of many countries are facing a number of problems such as lack of information and testing systems, insufficient and unqualified medical and nursing staff, and inadequate public health infrastructure, which are undoubtedly major factors in accelerating the spread of the epidemic. Equally worrying is the issue of government policy on epidemic prevention and control, and we believe that non-pharmaceutical interventions (NPIs) implemented by governments are equally important. It is clear from the global pandemic of COVID-19 that national infectious disease policies are more politically motivated than necessarily based on evidence (39). Reid-Henry divides the typology of global health policy and practice into two categories in his article: “social justice,” which focuses on reducing inequality, and “market justice,” which seeks to attain maximum utility. The former seeks to “maximize usefulness,” whereas the latter is focused on “reducing inequity (40).” China's epidemic prevention and control policies are developed and implemented with the lives and health of its citizens, social stability, and economic development in mind. Additionally, due to China's strong government power, it is able and willing to use a wide range of potent supply-oriented policy tools. However, it is clear that from the beginning of the COVID-19 pandemic, the United States oscillated between “controlling the epidemic” and “stabilizing the economy” with a distinct lack of unified national leadership and coordination, which led to a lack of strong plans (or common goals) from local and state health departments and the dissemination of confusing mixed messages to the lay public (41), resulting in a rapid spread of the epidemic until it was “out of control.” Sweden also has a very high COVID-19 mortality rate due to slow government response and weak policies (42). We agree with Ornelas that there is no “health versus economy” dichotomy (43). Some economists also demonstrated through their study that it would be irresponsible and unwise to forego containing the epidemic in order to realize short-term financial gain, as the estimated monetary value of lives saved by containing the virus is higher than the estimated loss of GDP caused by the lockdown (44). But the role of the government as the “bottom feeder” has been overlooked, even though in this day and age we emphasize the enormous power of the market and the irreplaceable role of the government, especially in crisis situations, where the serious effects of the crisis should not be borne entirely by the people.

In addition, more than 250 viruses have been transmitted from animals to humans and have led to pandemics (45). The novel coronavirus is an epidemic virus that humans are experiencing and will not be the last of its kind. Regional or global epidemics are expected to become the norm in a globalized world. Therefore, current outbreaks cannot simply be compared to those from the past. Scientists have also been emphasizing that the probability of new infectious diseases emerging is increasing exponentially (46) and that the answer to the question of whether the world is prepared for the next epidemic is probably no. So what steps should governments take to reduce or eliminate any potential risks?

First, the national health system needs to be optimized. Studies have shown that China responded to the COVID-19 outbreak more quickly than it did to the SARS outbreak in 2003, and it can be argued that the latter outbreak ultimately had a favorable effect on the structure of the Chinese health system by inducing the central government to refocus the CCDC and devote more funds to primary care (47). Countries should also fix the problems that public health systems have revealed in this epidemic and carry out system optimization. At the same time, it is important to focus on the huge role of public health institutions, as it was during the 1994 plague outbreak in Surat, India, when some 76% of private health sector practitioners fled the city (48). Under the COVID-19 epidemic, it was also public sector health care workers who were responsible for the control and treatment of the vast majority of infectious diseases. Second, governments must assume their fair share of responsibility in times of crisis. With a total of 3,856 social protection and labor measures planned or put into place in 223 economies as of January2022, including social assistance, supply-side labor market programs, and social insurance (49), countries are already actively working to lessen the impact of the pandemic on their peoples. The pandemic-related crises justify the interventionist approach and logic driven by the state welfare system, which supports the 'big government' model (50). The third is crisis readiness. This includes the preparation of medical supplies, the dissemination of knowledge on pandemic preparedness, and the encouragement of the use of advanced technologies such as artificial intelligence (AI) in the medical field, etc.

The public policy response to the COVID-19 crisis raises a number of questions that merit further investigation and debate, such as how to strike a balance between social isolation policies and economic development and how to adapt public policy in light of shifting epidemic conditions (including shifts in the public's perception of COVID-19 and the virus's ongoing mutation), etc. In a later study, it is hoped that these can be thoroughly examined.

## Conclusion

Public crisis management has already become one of the most essential regular duties in national governance, and using a combination of policy tools to transform public crises into peace is an unavoidable decision. On a global scale, many countries are still in the chronic phase of the COVID-19, and some are even in the outbreak phase. In China, only 3 months after the first case of COVID-19 was discovered, the government demonstrated their strong policy capabilities by effectively containing the spread of the pandemic and restoring the economic and social order to normal through a combination of supply-oriented, demand-oriented and environment-oriented policy tools.

## Data availability statement

The original contributions presented in the study are included in the article/supplementary material, further inquiries can be directed to the corresponding author.

## Author contributions

SZ decided on the theme of the study. SF and SZ drafted and revised the manuscript. XN was involved throughout the drafting and revision of the manuscript, helping to revise it repeatedly. YZ revised the manuscript repeatedly. All authors contributed to the article and approved the submitted version.

## Funding

The study was supported by Research on Urban Transportation Travel Considering Endogenous Spatial Effects Under Job-Housing Inequality: Impact Mechanisms, Empirical Tests and Policy Recommendations, 2022 Ministry of Education of China Humanities and Social Sciences Youth Foundation Project, Ministry of Education of the People's Republic of China, and Shanghai University Young Teacher Training Grant Program, University of Shanghai for Science and Technology, Shanghai, China.

## Conflict of interest

The authors declare that the research was conducted in the absence of any commercial or financial relationships that could be construed as a potential conflict of interest.

## Publisher's note

All claims expressed in this article are solely those of the authors and do not necessarily represent those of their affiliated organizations, or those of the publisher, the editors and the reviewers. Any product that may be evaluated in this article, or claim that may be made by its manufacturer, is not guaranteed or endorsed by the publisher.

## Footnotes

^1^The “four categories of persons” refer to confirmed patients, suspected patients, febrile patients and close contacts of confirmed patients. The “four types of persons” refer to those who should be admitted, treated, examined and isolated.

^2^The five hundred percent refers to hundred percent admission of confirmed patients, hundred percent nucleic acid testing of suspected patients, hundred percent testing of febrile patients, hundred percent isolation of close contacts, and hundred percent 24-hour closed management of small villages.

^3^The fourth edition was renamed the Pneumonia with Novel Coronavirus Prevention and Control guidelines and from the seventh edition onwards it was issued by the Joint Prevention and Control Mechanism of the State Council.

^4^Gansu Province, Liaoning Province, Guizhou Province, Yunnan Province and Guangxi Province have adjusted their response levels from Level 1 to Level 3; Shanxi Province, Guangdong Province and Jiangsu Province have adjusted their Level 1 responses to Level 2. The primary response is organized and coordinated by the provincial command authority in accordance with the central government's decision and unified command. The second level response is organized by the provincial command body to organize relevant units and personnel to study and judge the incident and then issue orders to all relevant units to start the relevant emergency procedures. Level 3 response is organized by the prefecture-level cities and counties (cities and districts) under the direct control of the province, and the relevant units and personnel will issue an order to the relevant units to start the relevant emergency procedures after the incident has been studied and judged.

^5^The circular published a total of 15 prevention and control technical plans for public places, shopping malls and playgrounds, public transport taxis, primary and secondary schools and other places.
